# Menstrual Health in Female Bodybuilders During Competition Preparation

**DOI:** 10.7759/cureus.89535

**Published:** 2025-08-07

**Authors:** Madison Lapp, Kendra Unger, Courtney Pilkerton, Erin White

**Affiliations:** 1 Orthopedics, West Virginia University, Morgantown, USA; 2 Family Medicine, West Virginia University, Morgantown, USA

**Keywords:** bodybuilding, low energy availability, menstrual health, nutrition and exercise, relative energy deficiency, women’s health

## Abstract

Introduction: Bodybuilding is a sport of self-discipline and pushing the human body to the extreme limits, often accomplished with drastic training methods and supplement usage, which have the potential to be associated with severe health consequences. Various aspects of bodybuilding competition preparation, such as nutrition, exercise, and drug/supplement utilization, contribute to changes in female athletes’ menstrual health.

Methods: An anonymous survey was conducted in women of reproductive age who were 18 and older assessing various techniques used during competition preparation and their menstrual cycles.

Results: Seven athletes completed the survey with the average weight loss during competition preparation being 6.4 kg. 42.9% of respondents reported using weight-cutting supplements and 28.6% reported using anabolic steroids. 85.7% of competitors had regular periods before competition and 57.1% experienced amenorrhea for at least one menstrual cycle during competition preparation.

Discussion: These competitors had low caloric intake for a prolonged period prior to competition, had high energy output, used exogenous supplements for cutting weight/augment training, and often experience amenorrhea for at least one menstrual cycle following competition.

Conclusion: While the sample size is limited, this study provides initial insight about an understudied population utilizing behaviors that have potential negative health outcomes when not conducted under the supervision of a healthcare professional.

## Introduction

Physique competitions, most commonly known as bodybuilding, are a lifestyle sport relying on strict discipline, nutrition, weightlifting, and cardiovascular exercise to obtain a desired body composition that is then subjectively judged and compared against other participants. Historically, bodybuilding has been a male sport, but with society now trending to value fitness, the desire for women to obtain a lean muscular physique has increased and promoted more female participation in bodybuilding. The criteria that have been developed for judging female physiques include muscularity and conditioning (that vary by division), symmetry and balance, and overall presentation [1]. Various competition divisions available for women include: bikini, figure, physique, wellness, bodybuilding, and fitness.

There is not a significant amount of peer-reviewed evidence available to guide nutrition and training practices of female competitors, so these athletes tend to rely on coaches who are considered experts in the world of bodybuilding. Coaches often obtain their knowledge and training strategies from trial-and-error practices with clients, or from non-peer reviewed online articles. Many of the most commonly used strategies (low energy intake, extreme energy expenditure, supplement usage, and water manipulation) to allow short-term benefit to the athlete to obtain her goal body composition but may have significant downstream health effects [2]. These health concerns include development of acute kidney injury, rhabdomyolysis, arrythmias, hypogonadism, thyroid dysfunction, and mood disorders.

Menstrual irregularities have a high prevalence in female athletes and are a part of the constellation of effects of low energy availability (LEA) leading to a condition previously termed as 'female athele triad'. However, in 2014, a consensus group recognized that the classic female athlete triad condition, which was characterized as LEA, amenorrhea, and osteoporosis, was not as inclusive of other affected health system due to LEA as it should be [3]. The group recognized that athletes also have increased risk of cardiovascular, psychosocial, musculoskeletal, and endocrine problems compared to their non-athlete peers. This understanding, coupled with the desire to include male athletes' health issues, prompted the 2014 International Olympic Committee (IOC) Consensus Statement, which coined a new term: relative energy deficiency in sport (RED-S). The IOC defined RED-S as a syndrome with “impaired physiological function including, but not limited to, metabolic rate, menstrual function, bone health, immunity, protein synthesis, cardiovascular health caused by relative energy deficiency”[3]. While sports with a high prevalence of eating disorders and high energy expenditures, such as running and cycling, are well known for predisposing athletes to menstrual irregularities, the sport of bodybuilding encourages the athlete to be in a prolonged state of energy deficiency with the goal of obtaining a low body fat percentage [4]. This places this subgroup of athletes at high risk of menstrual irregularities. The purpose of this study is to assess the effects of bodybuilding competition preparation (PREP), including nutrition, exercise, and drug/supplement utilization on female athletes’ menstrual health.

## Materials and methods

Female athletes who had recently participated in a bodybuilding competition were asked to complete an anonymous survey, including demographics, preparation for competition, and menstrual history (Appendix 1). The anonymous survey was granted exemption from the West Virginia University Institutional Review Board (210632533). The 17-question survey was created by the authors. It was conducted anonymously utilizing REDCap electronic data capture tool (StataCorp LLC, USA), which allows for secure data storage and export. The survey was distributed to potential participants via an advertising flyer posted on Instagram as well as through emails sent to multiple well-known National Physique Committee (NPC) bodybuilding coaches. It was further distributed utilizing snowball sampling. Inclusion criteria were women ages 18 and older who had competed in at least one bodybuilding competition within the three years of receiving the survey in 2021. A six-month period was allotted for survey completion with multiple reminders for completion and encouragement of snowball sampling.

The main outcome of interest was menstrual history. Menstrual-history questions included regularity and use of hormonal contraception prior to competition; menses missed during competition preparation, including last menstrual period prior to competition; as well as duration of return of menses post competition. 

Covariates of interest were age, current and previous competition categories, weight observations, diet observations (such as fat content and calorie counts), amount of cardiovascular training during majority of competition preparation, substance use related to weight, and menstrual history. Substance-use questions included use of anabolic steroids and use of cutting substances such as clenbuterol, Cytomel (T3), ephedrine-caffeine-aspirin (ECA), caffeine, nicotine, over-the-counter fat burners, and other substances. Nutritional history included caloric count and dietary fat amount for most of the competition preparation. Participants were also asked the source of their knowledge with regards to menstrual cycles during competition preparation. 

Data was analyzed using StataSE 16. Descriptive characteristics for each outcome and covariate were calculated (mean, percent, SD, etc). Due to low sample size, no statistical comparisons were made. 

## Results

Demographics 

For this study, 14 women started the survey and seven completed (50%). The average age of respondents was 29 with a range from 25 to 30 (Table 1).

**Table 1:** Descriptive characteristics of female bodybuilder competition preparation IUD: Intrauterine device; OCP: Oral contraceptive pill;  ECA: Ephedrine-caffeine-aspirin

	n (%)
Age (Mean (SD)	29 ± 4.3
Previous Classes Competed In	
Bikini	2 (28.6)
Figure	4 (57.1)
Wellness	1 (14.3)
Fitness	0 (0)
Physique	2 (28.6)
Bodybuilding	0 (0)
Most Recent Class Competed In	
Bikini	2 (28.6)
Figure	4 (57.1)
Wellness	0 (0)
Fitness	0 (0)
Physique	1 (14.3)
Bodybuilding	0 (0)
Average Weight before Competition (Mean (SD))	141 ± 17.5
Average Weight after Competition (Mean (SD))	127 ± 20.2
Regular Periods before Competition	
Yes	6 (85.7)
No	1 (14.3)
Skipped at Least One Period During Competition Preparation	
Yes	4 (57.1)
No	3 (42.9)
Types of Contraceptive Used	
None	2 (28.6)
Condoms	1 (14.3)
IUD	1 (14.3)
OCPs	3 (42.9)
Menstruation Information Source	
Internet	3 (42.9)
Forums	1 (14.3)
Books	1 (14.3)
Research Database	1 (14.3)
Healthcare Provider	2 (28.6)
Trainer	3 (42.9)
Friend	0 (0)
Other	3 (42.9)
Use of Substances to Aid Cutting Weight	
Anabolic steroid	2 (28.6)
Clenbuterol	1 (14.3)
Cytomel (T3)	1 (14.3)
ECA	1 (14.3)
Caffeine	4 (57.1)
Nicotine	0 (0)
Over-the-counter fat burners	2 (28.6)
Other	1 (14.3)
Target Calories Per Day During Competition Preparation	
<1,000	0 (0)
1,000 to 1,500	3 (42.9)
1,500 to 2,000	4 (57.1)
>2,000	0 (0)
Minutes of Cardiovascular Exercise During Competition Preparation Per Day	
<30	1 (14.3)
30 to 60	3 (42.9)
60 to 90	3 (42.9)
>90	0 (0)

Competition categories 

The majority of respondents had competed in the Figure category (n=4). The next most common competition categories were Bikini (n=2) and Physique (n=2). One respondent had competed in the Wellness category. The most recent bodybuilding classes these women entered were Figure (n=4), Bikini (n=2) and Physique (n=1). There were no competitors in the Fitness or Bodybuilding classes. 

Weight observations 

The average competitor weight prior to competition preparation was 64.1 kg. The average competitor weight following the cutting phase was 57.7 kg. The average weight loss during preparation for competition was 6.4 kg with a range of 3.2 to 9.5 kg. 

Dietary observations 

Three respondents maintained a daily calorie intake goal of 1,000 to 1,500 calories, while four respondents had an intake goal of 1,500 to 2,000 calories per day. These estimates were based on what an athlete felt they maintained for most of competition preparation. 

Cardiovascular training 

During most of the preparation, three competitors completed 60 to 90 minutes of cardiovascular exercise per day. Three competitors completed 30 to 60 minutes per day, and one competitor completed less than 30 minutes per day. 

Substance use related to weight 

Three respondents (42.9%) reported using weight reducing or “cutting” substances during training. One of the three respondents used multiple substances during cutting including Clenbuterol, T3, ECA, caffeine and over-the-counter fat burners. The most common substance used was caffeine (n=4). Two reported using over-the-counter fat burners. None of the competitors reported using nicotine. Two respondents (28.6%) indicated that they had used steroids to augments training.

Menstruation 

Six of seven competitors (85.7%) had regular periods before competition and four of seven (57.1%) experienced amenorrhea for at least one menstrual cycle during competition preparation. 

## Discussion

Menstrual irregularities have a higher prevalence among female athletes when compared to the average population. High physical demands and insufficient recovery, together with long-term inadequate nutritional intake and psychological stress are potential factors that cause an imbalance in the neuroendocrine process related to the hypothalamic-pituitary-ovarian (HPO) axis [5]. As stated previously, the origin of RED-S is a state of LEA. It is the nutritional energy deficit that directly leads to the other two hallmark symptoms observed in the female athlete triad: amenorrhea and osteoporosis. The signs of female athlete triad are just a small part of the RED-S that encompasses many other health systems that may include reduced immunity; impaired gastrointestinal, hematological, neurocognitive, cardiovascular, and skeletal muscle function; impaired growth and development; sleep disturbances; urinary incontinence; mental health issues; and the previously mentioned impaired reproductive function and bone health [6]. The involvement of these other health systems can have a major cardiovascular impact on the athlete. According to Berz and McCambridge, studies have demonstrated athletes with amenorrhea have a statistically significant decrease in flow-mediated dilation (FMD) of their brachial artery (by noninvasive ultrasound technique) compared to an athlete with oligomenorrhea or regular cycles [7]. A reduced FMD has been associated with an increased risk for atherosclerosis and is considered an early marker of cardiovascular disease. In addition, athletes who are amenorrheic have higher levels of cholesterol, apolipoprotein B, and low-density lipoprotein, which are risk factors for development of atherosclerosis. It is unclear if these abnormalities are reversible. 

The prevalence of the female athlete triad has been found in 1-14% of female athletes/performers, placing these individuals at risk for involvement of other health systems as seen in RED-S [7]. Menstrual irregularities are one of the more objective symptoms that can be measured. One study found that rhythmic gymnastics is a discipline in which the athletes suffer a major risk to present menstrual disorders, including 53.8%)for primary amenorrhea, 30.8% for secondary amenorrhea and 61% for oligomenorrhea [5]. In that same study by Gimunova et al., cyclic and individual sports disciplines also presented high frequencies of menstrual disorders, especially middle/long distance running (55% for secondary amenorrhea), cycling (55.6% for secondary amenorrhea), triathlon (40% for secondary amenorrhea), boxing (54.6% for oligomenorrhea), and tennis (42.9%) [5]. Our study focused on menstrual abnormalities in female bodybuilding finding similar rates to women in other sports with four of the bodybuilders (57.1%) surveyed reported oligomenorrhea/secondary amenorrhea. Chica-Lattore et al. conducted a meta-analysis to study the post-competition recovery phase [8]. Within the meta-analysis, 31 out of a total of 33 female athletes included in the literature experienced menstrual irregularities ranging from 10 to 71 weeks post contest. The participant whose menstrual function returned the quickest, also had the highest amount of energy intake post-contest. This further illustrates the relationship between LEA and menstrual irregularity. 

The sport of bodybuilding is founded on the premise of pushing the athlete’s body to the extremes of what the human body is able to achieve. Specifically, with regard to energy availability, one case study described a contest preparation in which the female participant reduced her daily calorie intake by 1,040 calories and lost 10.1 kg body weight (15% of starting body weight) over six months. This study observed a decrease in leptin and thyroid-stimulating hormone (TSH) consistently through the dieting phase. The athlete in this case study also experienced delayed menstrual cycles (four to five days) during the last two months of contest preparation and was not utilizing hormonal contraception [9]. The female bodybuilders surveyed by our study all acknowledged some form of LEA. This was achieved though caloric restriction, exercise, and use of certain substances. The average weight loss of these competitors was 6.4 kg, with a range of 3.2 kg to 9.5 kg. This weight loss was accomplished over a short period of time during the preparation phase. All competitors reported a goal of less than 2,000 calories per day while three (42.9%) maintained less than 1,500 calories. This deficit of energy due to restricted intake was further compounded by excessive cardiovascular training to further deplete energy stores. Only one competitor surveyed completed less than 30 minutes of cardiovascular exercise per day while the others completed at least 30 and some up to 90 minutes per day. In addition to caloric restriction and cardiovascular training, three (42.9%) further augmented this process by using “cutting substances", two using steroids and one using multiple substances. 

Our findings, while only on a small sample size, are consistent with other rates of menstrual irregularities seen in women competing in other sports that create a state of LEA. According to the Endocrine Society, the amenorrhea seen in athletes is termed functional hypothalamic amenorrhea, highlighting that there is not a dysfunction of the hypothalamus itself but rather the extreme behavioral changes in these athletes cause the dysfunction of the hypothalamic axis [10]. Our study demonstrated that female bodybuilders are at risk for developing RED-S and its consequences.

There are several limitations to this study. The main limitation is the small sample size of this survey. It was intended to obtain a much larger sample size; however, work in this population can have many barriers. Female bodybuilders are rare, and while this sport is growing in popularity, those who complete their training and compete are few as compared to their male counterparts. We hypothesize that the sensitive nature of the questions asked in this survey was also a deterrent for its completion (50% completion rate). While contributing to answers the questions posed would yield data that could strengthen recommendations for these competitors, many were likely concerned about answering these questions as they deal with the topics of substance use, menstruation, weight, and contraception. The paucity of response made reliable data analysis difficult and limited the ability to gain meaningful insight into the world of the female bodybuilder. Because of this, statistical analysis was not conducted and results were reported as descriptive data. More research is needed to enhance the performance of this rare, elite athlete. Another limitation is the self-report nature of our survey. Self-reported data from athletes often under-reports substance use and misreports diet habits [11,12]. While using self-reported data allows the participants to be anonymous, it may underestimate the highest risk health behaviors in an athlete’s training due to recall bias.

## Conclusions

There is very little research at the present time with regards to the female bodybuilder. Further information and guidelines made available to athletes, coaches, and medical providers can help bridge the gap and allow for a multi-disciplinary approach to optimize an athlete’s health and performance. While the bodybuilding population represents a small subset of athletes, and the female bodybuilder is rarer still, research in the sport has the potential to be extrapolated to the general population. Often, people in the general population succumb to extreme strategies, many listed above, to achieve a body type determined “desirable” by society. As exemplified by this research, these strategies are not well supported by data and pose significant health consequences.

## Appendices

Appendix 1

**Table 2:** Survey questions Credits: Madison Lapp, Courtney Pilkerton, Erin White, and Kendra Unger IUD: Intrauterine device; OCP: Oral contraceptive pill; ECA: Ephedrine-caffeine-aspirin

Please answer questions 3 through 15 regarding your most recent bodybuilding competition preparation.	
1. What is your age?	
2. In which divisions have you EVER competed? (Check all that apply)	
	a. Bikini
	b. Figure
	c. Wellness
	d. Fitness
	e. Physique
	f. Bodybuilding
3. In what division did you MOST RECENTLY compete?	
	a. Bikini
	b. Figure
	c. Wellness
	d. Fitness
	e. Physique
	f. Bodybuilding
4. What was your starting bodyweight during your last competition preparation?	
5. What was your bodyweight at the end of competition preparation?	
6. Were you having regular periods (every 25-31 days) before starting competition prep?	
	a. Yes
	b. No
7. Did you skip any menstrual cycles during competition preparation?	
	a. Yes
	b. No
8. How many weeks before the competition was the start of your last menstrual period?	
9. How many weeks after competition was the start of your first period post-competition?	
10. What type(s) of birth control, if any, were you using during competition preparation? (Check all that apply)	
	a. None
	b. Condoms
	c. Hormonal IUD
	d. Copper IUD
	e. OCPs
	f. Nexplanon or implant
	g. Transdermal patch
	h. Nuva ring
11. Did you use any anabolic steroids, such as Anavar, Winstrol, Testosterone, Primobolan, Nandrolone, etc., during competition preparation?	
	a. Yes
	b. No
12. Have you used other substances/drugs to aid with cutting during competition preparation? (Check all that apply)	
	a. Clenbuterol
	b. Cytomel (T3)
	c. ECA
	d. Caffeine
	e. Nicotine
	f. Over-the-counter fat burners
	g. Other
13. Approximately how many calories per day did you consume during the majority of your competition preparation?	
	a. < 1,000
	b. 1,000-1,500
	c. 1,500-2,000
	d. 2,000-2,500
	e. >2,500
14. Approximately how many grams of fat per day did you consume during the majority of your competition preparation?	
	a. < 20g
	b. 20-30g
	c. 30-40g
	d. 40-50g
	e. >50g
15. Approximately how many minutes per day did you engage in cardiovascular exercise during the majority of your competition preparation?	
	a. < 30 minutes
	b. 30-60 minutes
	c. 60-90 minutes
	d. 90-120 minutes
	e. >120 minutes
16. Have you ever looked up or been given information about menstrual cycles during training and competition?	
	a. Yes
	b. No
17. If you answered yes to the previous question, what was the source from where you got this information? (Check all that apply)	
	a. Internet
	b. Forums
	c. Books
	d. Research database
	e. Healthcare provider
	f. Trainer
	g. Friend
	h. Other
